# Long-term outcomes of left atrial appendage isolation using cryoballoon in persistent atrial fibrillation

**DOI:** 10.1093/europace/euac167

**Published:** 2022-09-27

**Authors:** Hikmet Yorgun, Yusuf Ziya Şener, Nikita Tanese, Ahmet Keresteci, Burak Sezenöz, Cem Çöteli, Ahmet Hakan Ateş, Serge Boveda, Kudret Aytemir

**Affiliations:** Faculty of Medicine, Department of Cardiology, Hacettepe University, Ankara 06230, Turkey; Department of Cardiology, Cardiovascular Research Institute Maastricht (CARIM), Maastricht University Medical Center+, Maastricht, The Netherlands; Faculty of Medicine, Department of Cardiology, Hacettepe University, Ankara 06230, Turkey; Department of Cardiology, Clinique Pasteur, Toulouse, France; Faculty of Medicine, Department of Cardiology, Hacettepe University, Ankara 06230, Turkey; Faculty of Medicine, Department of Cardiology, Gazi University, Ankara, Turkey; Faculty of Medicine, Department of Cardiology, Hacettepe University, Ankara 06230, Turkey; Faculty of Medicine, Department of Cardiology, Hacettepe University, Ankara 06230, Turkey; Department of Cardiology, Clinique Pasteur, Toulouse, France; Faculty of Medicine, Department of Cardiology, Hacettepe University, Ankara 06230, Turkey

**Keywords:** Atrial fibrillation, Left atrial appendage isolation, Pulmonary vein isolation, Cryoballoon

## Abstract

**Aims:**

There is an increasing trend evaluating the role of non-pulmonary vein (PV) triggers to improve ablation outcomes in persistent atrial fibrillation (AF) as pulmonary vein isolation (PVI) strategy alone has modest outcomes. We investigated the long-term safety and efficacy of left atrial appendage isolation (LAAi) in addition to PVI using cryoballoon (CB) in persistent AF.

**Methods and results:**

In this multicentre retrospective analysis, we included a total of 193 persistent AF patients (mean age: 60 ± 11 years, 50.3% females) who underwent PVI and LAAi using CB. Baseline and follow-up data including electrocardiography (ECG), 24 h Holter ECGs, and echocardiography were recorded for all patients. Atrial tachyarrhythmia (ATa) recurrence was defined as the detection of AF, atrial flutter, or atrial tachycardia (≥30 s) after a 3-month blanking period. At a median follow-up of 55 (36.5–60.0) months, 85 (67.9%) patients with PVI + LAAi were in sinus rhythm after the index procedure. Ischaemic stroke/transient ischemic attack occurred in 14 (7.2%) patients at a median of 24 (2–53) months following catheter ablation. Multivariate regression analysis revealed heart failure with preserved ejection fraction [hazard ratio (HR) 2.29, 95% confidence interval (CI) 1.04–5.02; *P* = 0.038], male gender (HR 0.53, 95% CI 0.29–0.96; *P* = 0.037), and LA area (HR 1.04, 95% CI 0.53–1.32; *P* = 0.023) as independent predictors of ATa recurrence.

**Conclusion:**

Our findings demonstrated that the LAAi + PVI strategy using CB had acceptable long-term outcomes in patients with persistent AF. Systemic thrombo-embolic events are an important concern throughout the follow-up, which were mostly observed in case of non-adherence to anticoagulants.

## Graphical Abstract

**Graphical Abstract:**
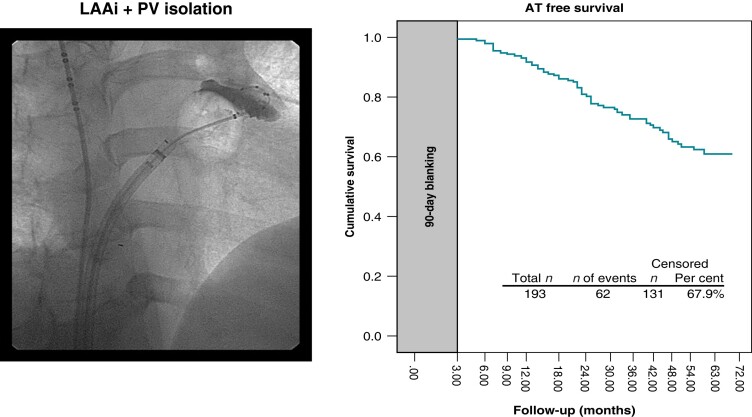

**What’s new?**

- Despite the favourable efficacy outcomes of left atrial appendage isolation (LAAi) in addition to pulmonary vein isolation (PVI), concerns regarding thrombo-embolic complications due to the decreased LAA contractile functions seem to be the main hurdle to implement this strategy to routine atrial fibrillation (AF) ablation procedure.
- We aimed to evaluate the long-term efficacy and safety outcomes of LAAi using cryoballoon as an adjunct to PVI in patients with non-paroxysmal AF.
- Our findings corroborated the previous data in terms of better efficacy of the PVI + LAAi strategy, whereas concerns regarding increased thrombo-embolic events should be considered.

## Introduction

Pulmonary vein isolation (PVI) is the key component of atrial fibrillation (AF) ablation procedure and first-line strategy in patients with paroxysmal and persistent AF.^1,2^ However, PVI has limited efficacy in patients with persistent AF mainly due to the atrial structural remodelling. Although ablation beyond PVI demonstrated no additional benefit over PVI-only approach, several recent studies highlighted the beneficial role of non-PV trigger ablation to improve procedural outcome, especially in patients with non-paroxysmal AF.^3–5^

Left atrial appendage (LAA) was shown to be a potential contributor to the initiation and maintenance of AF, which led to the emergence of left atrial appendage isolation (LAAi) in addition to PVI, using either radiofrequency (RF) or cryoballoon (CB) methods.^6,7^ In the randomized BELIEF trial, empirical LAAi had favourable outcomes in patients with long-standing persistent AF.^8^ Furthermore, improved outcomes with empirical LAAi strategy were also corroborated in CB ablation studies in persistent AF patients compared with PVI-only strategy without increasing acute procedural complications or systemic embolic event risk.^7,9^ Despite the favourable efficacy outcomes of LAAi, concerns regarding thrombo-embolic complications due to the decreased LAA contractile functions seem to be the main hurdle to implement this strategy in routine AF ablation procedure.^10,11^ Nevertheless, long-term follow-up data are scarce regarding the beneficial role of additional LAAi in persistent AF to improve catheter ablation outcomes as well as potential complications. In this multicentre study, we aimed to evaluate the long-term efficacy and safety outcomes of LAAi using CB as an adjunct to PVI in patients with non-paroxysmal AF.

## Methods

### Study population

In this retrospective analysis, we enrolled non-valvular symptomatic persistent AF patients who underwent the AF ablation procedure including both PVI and LAAi using the second-, third-, or fourth-generation CB between January 2015 and March 2020 in two centres [Hacettepe University, Department of Cardiology, Ankara Turkey (158 patients) and Clinic Pasteur, Toulouse, France (35 patients)]. Persistent AF was defined as AF duration >7 days and <1 year, whereas long-standing persistent AF was defined as AF duration ≥1 year if the rhythm control strategy is adopted.^1,2^

The medical history of the enrolled was taken from the hospital records. The exclusion criteria were as follows: uncontrolled thyroid dysfunction, thrombus in the left atrium (LA), severe valvular disease, pre-procedural significant coronary artery disease, myocardial infarction or cardiac surgery in the previous 3 months, previous atrial tachyarrhythmia (ATa) ablation history, pregnancy, postero-anterior LA diameter of >55 mm, and life-expectancy <12 months.

The study was in compliance with the principles outlined in the Declaration of Helsinki and approved by Institutional Ethics Committee.

### Pre-procedural management

Pre-procedural transthoracic echocardiography (TTE) was performed in all patients. Transoesophageal echocardiography (TEE) was performed before the procedure to evaluate LA thrombus and LAA flow velocities. Multidetector cardiac computed tomography was performed to assess LA and PV anatomy. All anti-arrhythmic drugs (AADs) were ceased 5 half-lives before the procedure except amiodarone. Novel oral anticoagulants (NOACs) were temporarily ceased 24–48 h before the procedure according to the patient’s glomerular filtration rate. Since the latest AF ESC Guidelines, NOACs were uninterrupted during the procedure.

### Atrial fibrillation ablation using cryoballoon

The details of AF ablation procedure including PVI and LAAi were described previously.^9^ Briefly, AF ablation was performed under either conscious or deep sedation/general anaesthesia. After femoral vein punctures, a 6 Fr steerable multipolar catheter was placed into the coronary sinus. Single transseptal puncture by modified Brockenbrough technique was performed under fluoroscopy. Unfractionated heparin boluses were administered to maintain the activated clotting time (ACT) of 300–350 s, after LA access was obtained. The steerable FlexCathAdvance sheath (Medtronic CryoCath, Minneapolis, MN, USA) was placed into the LA. A 28 mm CB catheter (Arctic Front Advance™, Arctic Front Advance ST, or Artic Front Advance Pro, Medtronic, Minneapolis, MN, USA) was used for PVI. Pulmonary vein potentials were recorded using the 15/20 mm Achieve catheter (Achieve™, Medtronic, Minneapolis, MN, USA). The duration of each freezing cycle was 180–240 s and bonus freeze was applied if PV potentials disappeared >60 s of the freeze cycle. Phrenic nerve palsy (PNP) was assessed by direct palpation of right hemidiaphragmatic movement under constant pacing from the superior vena cava during freezing at the right-sided PVs.

After isolation of all PVs, Achieve™ catheter was placed inside the LAA and occlusion was checked with manual contrast injection after inflation of the CB at the LAA ostium. In the first 20 patients, the duration of CB freeze was 450 s and was set to 240–300 s subsequently. Bonus freeze was applied if LAAi could not be achieved in 150 s. The integrity of the left PN was assessed by pacing either from the LAA using Achieve™ catheter or from the left subclavian vein using a decapolar catheter throughout the freezing cycle. Coronary angiography was performed after LAAi isolation if needed to rule out coronary artery vasospasm.

Successful PVI or LAAi was defined as the elimination (or dissociation) of the potentials recorded by Achieve catheter. The entrance and exit blocks were confirmed by pacing manoeuvres using a coronary sinus electrode or circular mapping catheter when needed. Due to the possibility of early reconnection, a waiting period of at least 15 min was considered and reablation was performed if reconnection occurred. The figure-of-eight suture technique was used for the 15 Fr venous sheath as described before.^12^

### Re-do procedure and percutaneous left atrial appendage occlusion

Re-do catheter ablation procedures for the recurrence of atrial tachycardias (ATs) were performed with RF ablation by using (3D) mapping systems, either by Carto (Biosense Webster, USA) or Ensite Precision System (Abbott, USA). A baseline electrophysiologic study was performed before the evaluation of PV or LAA reconnection.

Reconnection was defined as the presence of electrical conduction between LA and corresponding PVs or LAA. Radiofrequency ablation was performed with a 3.5 mm contact-force sensing irrigated-tip catheter (SmartTouch Catheter, BiosenseWebster, or TactiCath Ablation Catheter, Abbott). If LAA or PV electrical reconnection was detected, focal ablation was performed at the reconnection gap. In case of isolated PVs and LAA, other non-PVI foci were evaluated under isoproterenol infusion (up to 20 µg/min). Substrate modification via complex fractionated atrial electrogram ablation or linear lesion formation was performed in the presence of low-voltage areas. In case of AT, activation mapping and entrainment manoeuvres were performed to determine the tachycardia mechanism. After the termination of the tachycardia by RF ablation, non-inducibility was checked by programmed stimulation.

Percutaneous LAA occlusion was performed in patients with previous AF-related ischaemic stroke, severe smoke in LAA/decreased LAA flow velocities, or in patients who were ineligible to use oral anticoagulant (OAC) due to major bleeding during the follow-up. The Amplatzer Amulet LAA occlusion device (Abbott Vascular) was used for LAA occlusion.

### Post-procedural management and follow-up

Oral anticoagulation was continued 6 h after the procedure if there was no bleeding complication. Follow-up visits were scheduled at 3, 6, and 12 months and every 12 months thereafter or earlier if the patient had symptoms consistent with recurrent ATa or procedure-related complication was suspected. The 12-lead electrocardiography (ECG) and TTE were performed at each follow-up visit. Transoesophageal echocardiography was scheduled at 3rd and 12th month visits after ablation to assess LAA flow velocity, degree of smoke, or thrombus formation. A 24 h Holter ECG was recorded in the 3rd month and yearly after the procedure. Continuation of AADs beyond 3 months after ablation was left at the discretion of the attending physician. Independent of the CHA_2_DS_2_-VASc score, all patients were strictly advised to use life-long OAC after the ablation procedure.

### Study endpoints

Procedural success was defined as electrical isolation of all PVs and LAA. A blanking period was defined for the first 3 months after the AF ablation. Atrial tachyarrhythmia recurrence was defined as the detection of AF or AT (≥30 s) with Holter monitoring or surface ECG. Any recurrence within the first 3 months of ablation was defined as early recurrence, whereas recurrence >3 months was defined as late recurrence. Freedom from ATa recurrence at the last follow-up visit was the primary endpoint of the study. All complications including ischaemic stroke/transient ischemic attack (TIA), bleeding, and death were recorded throughout the study period.

### Statistical analysis

Continuous variables with normal distribution were presented as mean values ± SD and median (minimum–maximum) was used for non-parametric variables. Categorical variables were presented as number (*n*) and percentage (%). The detrended Q–Q plot and Kolmogorov–Smirnov tests were used for the assessment of normality. Comparisons between variables were performed by the Mann–Whitney *U* rank-sum, independent Student’s *t*-test, *χ*^2^ test, and Fisher exact test where appropriate. The Cox proportional hazards regression was preferred for determining factors related to AT recurrence and variables in association with recurrence with *P* < 0.2 in univariate regression analysis were included in multivariable Cox regression analysis. The Kaplan–Meier curve was used to demonstrate freedom from TIA/stroke or ATa recurrence within years. A two-tailed *P*-value of <0.05 was defined as statistically significant.

## Results

### Study population

Among a total of 212 persistent AF patients in whom PVI + LAAi was attempted, complete LAAi could not be achieved in 19 (8.9%) patients (in 15/19 patients, ablation was not performed due to incomplete occlusion, in 4/19 of them electrical connection persisted despite good occlusion). Finally, 193 patients were enrolled [97 (50.3%) patients were female, mean age 60 ± 11 years]. The median duration of AF was 24 months and 43.5% of the patients underwent electrical cardioversion before admission. The mean left ventricular ejection fraction (LVEF) was 59.5 ± 8.3% and the mean LA area was 23.7 ± 8.2 cm^2^. Baseline characteristics of the patients are presented in *Table 1*.

**Table 1:** Baseline characteristics of the study population (*n* = 193)

Age (years)	60.5 ± 11.1
Gender (female)	97 (50.3%)
Comorbidities	
ȃHypertension	102 (52.8%)
ȃDiabetes mellitus	38 (19.7%)
ȃCoronary artery disease	35 (18.1%)
ȃHeart failure	39 (20.2%)
ȃHFrEF	20 (10.4%)
ȃHFpEF	19 (9.8%)
ȃStroke	9 (4.7%)
Medications	
ȃRenin–angiotensin system blockers	76 (39.4%)
ȃBeta-blockers	110 (57%)
ȃCalcium channel blockers	27 (14%)
ȃDigoxin	20 (10.4%)
ȃAnticoagulant drug	137 (69.4%)
ȃȃVKA	30 (15.5%)
ȃȃNOAC	107 (55.4%)
ȃȃȃRivaroxaban	59 (30.9%)
ȃȃȃApixaban	27 (14.0%)
ȃȃȃDabigatran	15 (7.8%)
ȃȃȃEdoxaban	6 (3.1%)
ȃȃAADs	95 (49.2%)
ȃȃȃPropafenone	35 (18.1%)
ȃȃȃFlecainide	5 (2.6%)
ȃȃȃAmiodarone	47 (24.4%)
ȃȃȃSotalol	8 (4.1%)
Echocardiographic parameters	
ȃLV EDD, mm	47.9 ± 5.5
ȃLVEF, %	59.5 ± 8.3
ȃLA area, cm^2^	23.7 ± 8.2
ȃMitral regurgitation	
ȃȃModerate	64 (37.4%)
ȃȃModerate–severe	3 (1.8%)
ȃsPAP, mmHg	34.1 ± 8.7
AF characteristics	
ȃType of AF	
ȃȃPersistent	163 (84.5%)
ȃȃLong-standing persistent	30 (15.5%)
ȃPrior DCCV	84 (43.5%)
ȃDuration of AF history (months)	24 (6.5–50)
CHAD_2_S-VA_2_Sc	2 (1–3)
HAS-BLED	1 (0–5)
EHRA Class	2 (2–2)

AAD, anti-arrhythmic drug; AF, atrial fibrillation; DCCV, direct current cardioversion; EHRA, European Heart Rhythm Association; GFR, glomerular filtration rate; HfpEF, heart failure with preserved ejection fraction; HfrEF, heart failure with reduced ejection fraction; IVS, interventricular septum; LA, left atrium; LAA, left atrial appendage; LV EDD, left ventricular end-diastolic diameter; LVEF, left ventricular ejection fraction; NOAC, novel oral anticoagulants; sPAP, systolic pulmonary artery pressure; VKA, vitamin K antagonist.

### Procedural characteristics

The total procedural time was 62.9 ± 7.0 min and the total fluoroscopy time was 7.2 ± 0.8 min. The mean number of CB applications per PV was 1.53 ± 0.58. The acute procedural success rate for PVI was 98.9%. Cryoballoon for LAAi was applied for a mean 1.27 ± 0.48 cycles, median time to isolation was 123 (33–370) s, and median temperature at isolation was −41°C (−28 to −58°C). Detailed procedural characteristics are shown in *Table 2*.

**Table 2:** Procedural characteristics of the study population

Total procedure time (min)	62.9 ± 7.0
Fluoroscopy time (min)	7.2 ± 0.8
Mean no. of freeze–thaw cycles	1.53 ± 0.58
Left superior PV	
ȃTemperature at isolation (°C)	−37.5 ± 2.6
ȃNadir temperature (°C)	−47.2 ± 5.5
ȃTime-to-isolation (s)	45.5 (15–88)
Left inferior PV	
ȃTemperature at isolation (°C)	−34.0 ± 2.88
ȃNadir temperature (°C)	−45.0 ± 4.6
ȃTime-to-isolation (s)	42 (19–82)
Right superior PV;	
ȃTemperature at isolation (°C)	−32.6 ± 5.0
ȃNadir temperature (°C)	−46.6 ± 3.97
ȃTime-to-isolation (s)	42 (12–82)
Right interior PV	
ȃTemperature at isolation (°C)	−32.6 ± 4.9
ȃNadir temperature (°C)	−49.9 ± 4.42
ȃTime-to-isolation (s)	47 (18–84)
Left atrial appendage	
ȃMean no. of freeze–thaw cycles	1.27 ± 0.48
ȃTime-to-isolation (s)	123 (33–370)
ȃTemperature at isolation (°C)	−41 (28–58)
ȃNadir temperature (°C)	−53 (35–64)
ȃTotal freezing time (s)	310 (290–455)

Successful LAA closure was performed in a total of seven patients. Indications for LAA occlusion were gastrointestinal bleeding under OAC therapy in one patient and intracranial bleeding in two patients. In four patients, LAA closure was performed due to the presence of severe smoke with significantly decrease in LAA emptying velocity.

### Complications

Access site complications including inguinal haematoma and pseudoaneurysm occurred in seven (3.6%) patients and resolved without surgical intervention. Mild to moderate pericardial effusion developed in five (2.6%) patients, and a pericardiocentesis was required in only one (0.5%) patient. Phrenic nerve damage occurred in seven (3.6%) patients [right PNP in five (2.6%) patients; left PNP in two (1%) patients]. Among right PNPs, two out of five were transient and three out of five were persistent, whereas all left PNPs were persistent; however, they all resolved during the follow-up. Left circumflex artery spasm occurred in six (3.1%) patients and all of them were resolved with intracoronary nitrate infusion.

### Thrombo-embolic and bleeding events

**Figure 1:**
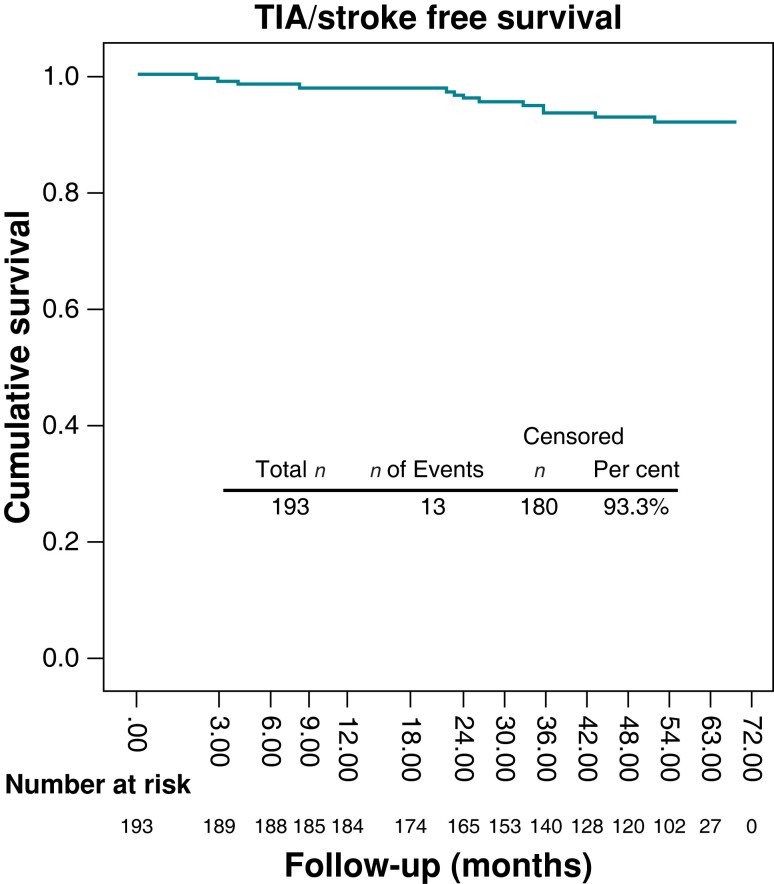
Kaplan–Meier analysis illustrating freedom from TIA/stroke (92.8%) strategies during the median follow-up of 55 (36.5–60.0) months.

In-hospital ischaemic stroke or TIA did not occur in any patient. Systemic thrombo-embolic events developed in 14 (7.2%) patients during the follow-up period (ischaemic stroke in 11 patients, TIA in 2 patients, retinal artery embolism in 1 patient). The median time of cerebrovascular event occurrence was 24 (2–53) months after catheter ablation. Freedom from TIA/ischaemic stroke is depicted in *Figure 1*.

Left atrial appendage emptying velocity was still lower in patients with thrombo-embolism than counterparts (0.28 ± 0.06 m/s vs. 0.43 ± 0.14; *P* < 0.001) at 12th month control TEE.

All of the patients experiencing ischaemic stroke had a CHA_2_DS_2-_VASc score ≥ 2 and LAA flow velocity of <0.4 m/s at the 12th month of control. The mean LAA emptying velocity was significantly lower in patients with systemic thrombo-embolism compared with the patients without thrombo-embolic events (0.28 ± 0.06 vs. 0.44 ± 0.15; *P* < 0.001) at the median 12th month (10–15) control. Despite the advices for strict adherence to OAC therapy, all ischaemic stroke events were due to the interruption of anticoagulation therapy (13/14 non-adherence to OAC regimen and 1/14 total cease of OAC) (see Supplementary material online).

Stroke-related death occurred in one patient, and six patients recovered from stroke with variable sequelae in motor functions with the median Modified Rankin Score of 1 (0–3) in these patients. Left atrial appendage emptying velocity was significantly reduced during follow-up in patients with TIA/stroke compared with baseline levels (0.56 ± 0.17 vs. 0.28 ± 0.06 m/s; *P* = 0.044). In four patients, thrombus in the LAA was observed which resolved with intensifying or optimizing OAC therapy. There was a significant increase in the percentage of patients on anticoagulation (NOACs or warfarin) (55.4% before ablation vs. 77.2% in the last visit, *P* < 0.001).

### Procedural outcomes

**Figure 2:**
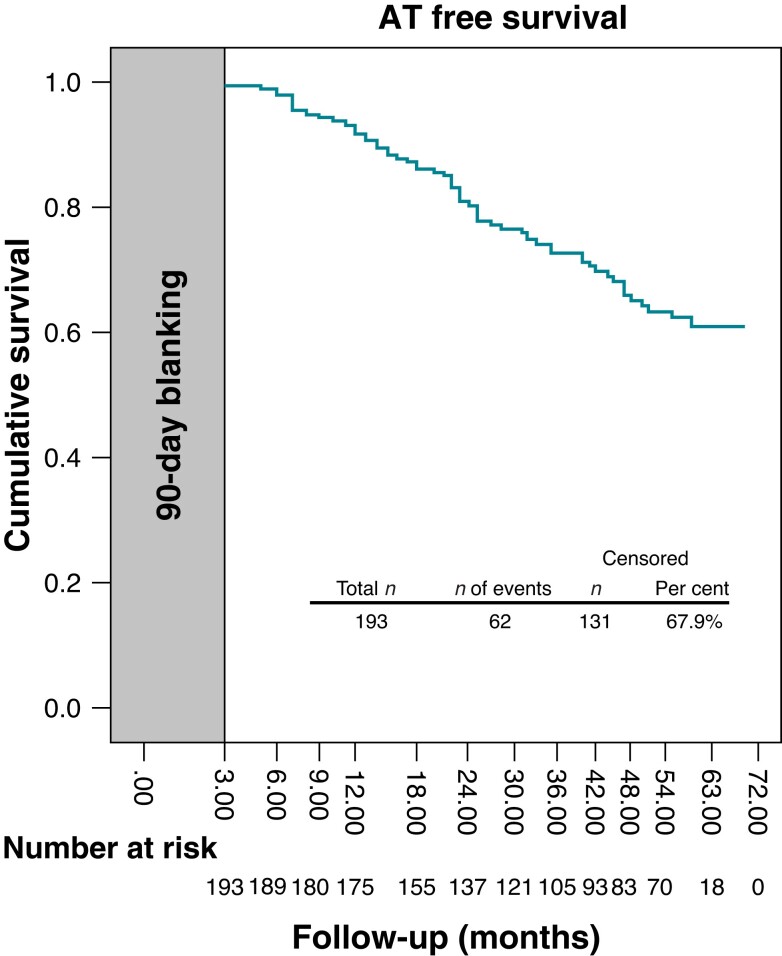
Kaplan–Meier analysis illustrating freedom from ATa after PVI + LAAi approach during the median follow-up of 55 (36.5–60.0) months after exclusion of blanking period (90 days after ablation). AT, atrial tachycardia.

The median follow-up duration was 55 (36.5–60.0) months. Early recurrence was observed in 14 (7.2%) patients. Atrial fibrillation was the most common (12/14) type recurrence, while left AT (1/14 patients) and typical isthmus-dependent atrial flutter (1/14 patients) was detected in the remaining two patients. Late recurrence occurred in 62 (32.1%) patients. In the 14 patients with early recurrence, 4 of them also had late recurrence. The median time to late recurrence was 22.5 (3–59) months. Atrial tachyarrhythmia–free survival at the 1st, 2nd, and 3rd year was 92.8, 79.5, and 70.6%, respectively. During the long-term follow-up, 67.9% of the patients were free of ATa after the CB-based PVI and LAAi (*Figure 2*). Late recurrence rates were not different between the patients with and without AAD administration at discharge [53 (33.9%) vs. 9 (24.3%), *P* = 0.350].

The most common type of late recurrence was AF in 45 of 62 patients, whereas left AT was detected in 10 of 62 patients and cavotricuspid isthmus-dependent (CTI) atrial flutter was detected in 5 of 62 patients. In two patients, right AT was detected. The outcomes of the study population are presented in *Table 3*.

**Table 3:** Outcomes of LAAi and PVI during long-term follow-up (*n* = 193)

Parameters	PVI and LAAi group (*n* = 193)
Median follow-up (months)	55 (36.5–60.0)
Early recurrence	14 (7.2%)
Late recurrence	62 (32.1%)
Thrombo-embolic events, *n* (%)	
ȃStroke	11 (5.6%)
ȃTIA	2 (1.0%)
ȃRetinal artery embolism	1 (0.05%)
Bleeding events	
ȃMajor bleeding	5 (2.6%)
ȃCRNM bleeding	9 (4.7%)
All-cause mortality	10 (5.2%)

CRNM, clinically relevant non-major bleeding; LAAi, left atrial appendage isolation; PVI, pulmonary vein isolation; TIA, transient ischaemic attack.

The mortality rate during follow-up was 5% (10/193) in the whole study population which was due to malignancy (2/10), sepsis (2/10), stroke (1/10), and coronary artery disease (1/10). In 4 of 10 patients, the cause of death was not reported in the electronic database or derived from the patients’ files. No death could be directly linked with the ablation procedure.

### Findings in re-do patients

Among patients with late recurrence, 34 (54.8%) patients underwent repeat catheter ablation procedure. Reconnection at LAA was observed in 8 of 34 (23.5%) patients. Furthermore, CTI-ablation due to typical atrial flutter (*n* = 8), anterior line (*n* = 9), posterior mitral isthmus line (*n* = 6), roof line (*n* = 8), left atrial scar modification (*n* = 14), alcohol ablation of Marshall ligament (*n* = 2), crista terminalis and SVC ablation (*n* = 1), slow pathway ablation for AVNRT (*n* = 2), and right focal AT (*n* = 2) ablations were also performed. Furthermore, in three patients with durable LAA electrical isolation, dissociated LAA automaticity was observed.

### Predictors of recurrence

Univariate Cox proportional hazard analysis showed that heart failure with preserved ejection fraction (HFpEF) [hazard ratio (HR) 2.61, 95% confidence interval (CI) 1.32–5.16; *P* = 0.006], male gender (HR 0.66, 95% CI 0.40–1.11; *P* = 0.0121), AF duration (HR 1.00, 95% CI 0.99–1.00; *P* = 0.193), history of DCCV (HR 1.50, 95% CI 0.91–2.48; *P* = 0.109), beta-blocker usage (HR 1.44, 95% CI 0.86–2.41; *P* = 0.159), CHA_2_DS_2_-VASc score (HR 1.12, 95% CI 0.94–1.34; *P* = 0.179), LA area (HR 1.04, 95% CI 1.01–1.07; *P* = 0.006), and severity of mitral regurgitation (HR 1.34, 95% CI 0.94–1.90; *P* = 0.100) were predictors of ATa recurrence. Multivariate regression analysis revealed that HFpEF (HR 2.29, 95% CI 1.04–5.02; *P* = 0.038), male gender (HR 0.53, 95% CI 0.29–0.96; *P* = 0.037) and LA area (HR 1.04, 95% CI 0.53–1.32; *P* = 0.023) were determined as independent predictors of ATa recurrence (*Table 4*).

**Table 4:** Univariate and multivariate Cox proportional Hazard modelling results of the ATa recurrence after CB-based AF ablation

	Univariate analysis			Multivariate analysis		
	HR	0.95 CI	*P*-value	HR	0.95 CI	*P*-value
HFpEF	2.61	1.32–5.16	0.006	2.29	1.04–5.02	0.038*
Gender, male	0.66	0.40–1.11	0.121	0.53	0.29–0.96	0.037*
Duration of AF	1.00	0.99–1.00	0.193	1.00	1.00–1.01	0.050
DCCV	1.50	0.91–2.48	0.109	1.25	0.71–2.19	0.432
Beta-blocker	1.44	0.86–2.41	0.159	1.94	0.91–2.94	0.094
CHA_2_DS_2_-VASc	1.12	0.94–1.34	0.179	0.93	0.76–1.15	0.546
LA area	1.04	1.01–1.07	0.006	1.04	1.00–1.08	0.023*
MR	1.34	0.94–1.90	0.100	0.83	0.53–1.32	0.447

*Statistically significant. AF, atrial fibrillation; DCCV, direct current cardioversion; HfpEF, heart failure with preserved ejection; LA, left atrium; MR, mitral regurgitation.

## Discussion

The results of this study were comparable with previous studies^13^ evaluating PVI + LAAi approach in persistent AF patients and demonstrated an ATa-free survival of 67.9% at a median of 55 months. Among several variables, HFpEF, male gender, and LA area were independent predictors of ATa recurrence. The incidence of systemic thrombo-embolic events was 7.2%, which mainly occurred at a median of 24 months after ablation. Beyond the sustained long-term efficacy outcomes of LAAi, these findings highlight the concerns regarding thrombo-embolic complications attributable to the LAAi, which necessitate the consideration of optimal patient selection as well as potential strategies to reduce future adverse event risk.

The key role of PVI in catheter ablation of AF has long been known; however, ATa-free survival is modest in persistent type compared with the paroxysmal AF.^3,14,15^ Several previous studies showed the role of additional potential sources that can be targeted during ablation.^16–18^ Among these, the clinical benefit of additional LAAi in addition to PVI in persistent AF patients was also demonstrated.^6,19^ In the BELIEF study, empirical LAAi had better AF ablation outcomes compared with an extensive ablation without LAAi at 24th-month follow-up in patients with long-standing persistent AF.^8^ Furthermore, Romero *et al*.^13^ reported higher freedom from ATa recurrence off-AADs in patients who underwent LAAi was compared with the standard ablation group at 5-year follow-up (68.9 vs. 50.2%, respectively). Using CB technology-only, our ∼5-year follow-up findings revealed similar freedom from ATa. These findings emphasize the efficacy of LAAi as an adjunct to PVI, irrespective of the ablation tool used.

Our study expands the literature specifically in terms of the number of patients enrolled as well as the longest follow-up duration to evaluate the outcomes of CB-based LAAi strategy. Our group previously demonstrated the role of empirical LAAi in addition to PVI using CB for the first time in persistent AF, which revealed a significantly improved 1-year outcome of 86% compared with PVI-alone approach.^7^ Furthermore, improved outcomes with LAAi in addition to PVI were maintained (ATa-free survival: 75.7%) compared with a propensity-matched PVI-only group without an increase in the thrombo-embolic event rate during a longer follow-up of 30.5 ± 5.6 months.^9^ These findings were also consistent with previous RF-based LAAi studies demonstrating improved efficacy of LAAi to improve outcomes in patients with persistent AF.^20^ Beyond favourable early and mid-term outcomes, the results of this long-term follow-up study demonstrated sustained beneficial effects of LAAi in addition to PVI to improve ATa-free survival after an index CB ablation procedure. On the other hand, in a recent study by Tohoku *et al*.^21^ despite straightforward LAAi using CB, freedom from ATa was higher after RF-based wide-area isolation. These results might be due to the ablation of additional targets by creating lines and more substrate modification using RF compared with the CB tool that only aimed LAA as non-PV trigger as well as different patient characteristics or extent of atrial underlying substrate causing arrhythmogenesis.

Although additional LAAi seems to improve freedom from ATa recurrence, the net clinical benefit of LAAi is still a matter of debate because of empirical ablation approach without the demonstration that the LAA was the trigger for AF. Although initial studies reported a higher incidence of LAA as AF trigger, this was not found as high in a recent study which is mostly due to the definition of non-PV trigger.^22^ Furthermore, reconnection following LAAi via CB application is not an uncommon finding which was reported in 23.5% of our re-do procedures. Despite these limitations, the benefit of empirical LAAi might be due to several factors including (i) LAAi itself; (ii) better left lateral ridge ablation and Marshall ligament ablation; and (iii) substrate ablation in the osteal part of LAA which was shown to be associated with localized re-entries.^19^ Further studies are needed to elucidate the role of LAAi in this patient group.

Systemic thrombo-embolism is the most feared complication of electrical LAAi. In a previous study with a long-term follow-up of 5 years, thrombo-embolic events occurred in 2.75% patients in the LAAi group, whereas 0.73% in the non-LAAi group.^13^ In this study, the median time to stroke was not significant between the LAAi and non-LAAi groups (21.4 months in LAAi and 22.3 months in the non-LAAi groups). Similar to these findings, the median time to systemic thrombo-embolic events was 24 months in our study group. However, our findings indicated a higher percentage of ischaemic thrombo-embolic events (7.5%), at a similar follow-up duration. These findings might highlight the safety concerns regarding late thrombo-embolic events after ablation possibly due to the progressive atrial remodelling as well as worsening in LAA functions caused by isolation procedure itself. Furthermore, in four of our patients with a decreased appendage flow velocity after isolation, control TEE during follow-up revealed thrombus in the LAA which resolved after intensification of OAC regime. These findings may imply for routine TEE screening even in asymptomatic patients if LAA closure was not scheduled. Although anticoagulation adherence was improved at the final visit compared with the baseline level, undertreatment rate was still high, which seemed to be a major determinant for thrombo-embolic complications. Beyond these, achievement of sinus rhythm after LAAi might cause underutilization of medications related to AF. On the other hand, a recent report highlighted the importance of LAA closure to prevent thrombo-embolic events despite oral anticoagulation therapy in patients with both RF- or CB-based LAAi.^23^ The difference in the thrombo-embolic event rate despite OACs may be due to the type of oral anticoagulation, basal characteristics of the study population, as well as the extent of unhealthy atrial underlying substrate. As a result, early LAA closure should be considered in order to prevent future thrombo-embolic events compared in case of non-adherence with OAC therapy.

Procedural complications during LAAi due to the anatomical close proximity of LAA ostium with coronary arteries and left phrenic nerve are not uncommon. Circumflex artery spasm was reported in 6 (3.1%) in our patient group, which were all resolved after intracoronary nitrate injection. In addition, left PNP is another concern during LAAi which was reported in 1% of our patients. Therefore, specific complications related to LAAi should be kept in mind in addition to PVI-related adverse events.

Despite the strengths of our study findings, this study has several limitations. Although including the highest number of persistent AF patients ablation with LAAi using CB, this was a retrospective analysis, which limits the clinical relevance of our findings. Secondly, the control group was not present to directly compare the safety and efficacy of additional LAAi with PVI-only approach. Thirdly, we were unable to perform TEE on every patient during the follow-up which might limit to demonstrate the exact physiologic effects of LAAi. Finally, recovery of connection after LAAi is a common phenomenon which might create a hurdle to attribute the clinical benefit only to LAAi procedure itself.

In conclusion, in our experience, LAAi in addition to PVI was associated with acceptable long-term outcomes which were maintained during long-term follow-up. Although observed in patients with poor anticoagulation adherence, thrombo-embolic events after LAAi were remarkable, which reinforces the need for interventional LAA closure after such a procedure. Long-term randomized studies are needed to elucidate the safety and efficacy of empirical LAAi along with PVI in the management of AF.

## Supplementary Material

euac167_Supplementary_Data

## Data Availability

Data available on request.
