# Supplementary material for: Long-term outcomes of left atrial appendage isolation using cryoballoon in persistent atrial fibrillation
Source: Europace. 2022 Sep 27;25(2):366–73. doi: 10.1093/europace/euac167 (PMC10103563; doi:10.1093/europace/euac167)
Supplement: euac167_Supplementary_Data [file euac167_supplementary_data.zip › Author aggr form.pdf]

Author Declaration Form

Title of Manuscript:

Long term Outcomes Left Atrial Appendage Isolation using Cryoballoon in Persistent Atrial Fibrillation

This manuscript, or part of it, has neither been published nor is currently under consideration by any other Journal.

I declare that:

(*Please tick the relevant statement below*):

☐ my co-authors listed below have read the manuscript and approved its submission to Europace. Each author has signed to that effect below, or

☒ in the case of 5 or more authors, every author has read and approved the manuscript and has delegated me, as corresponding author, to sign this declaration on their behalf.

Printed corresponding author name	Corresponding author signature
Hikmet Yorgun	[signature]

Printed Co-author name	Co-author signature
Yusuf Ziya Kılıç	
Nikita Tanese	
Ahmet Keresteci	
Burak Sezenöz	

Cem Çöteli	
Ahmet Hakan Ateş	
Serge Boveda	
Kudret Aytemir	
