# Supplementary material for: Long-term outcomes of left atrial appendage isolation using cryoballoon in persistent atrial fibrillation
Source: Europace. 2022 Sep 27;25(2):366–73. doi: 10.1093/europace/euac167 (PMC10103563; doi:10.1093/europace/euac167)
Supplement: euac167_Supplementary_Data [file euac167_supplementary_data.zip › Supplementary Table new.docx]

**Supplementary Table:**

| **Causes of drug incompliance in patients with systemic thromboembolism (n=14)** | |
| --- | --- |
| Forgetting to take the drug due to polypharmacy | 7 (50%) |
| Bleeding episode | 6 (43%) |
| Absence of health insurance | 1 (7%) |
